# Targeting PSMA Revolutionizes the Role of Nuclear Medicine in Diagnosis and Treatment of Prostate Cancer

**DOI:** 10.3390/cancers14051169

**Published:** 2022-02-24

**Authors:** Wietske I. Luining, Matthijs C. F. Cysouw, Dennie Meijer, N. Harry Hendrikse, Ronald Boellaard, André N. Vis, Daniela E. Oprea-Lager

**Affiliations:** 1 Department of Urology, Prostate Cancer Network Netherlands, Amsterdam University Medical Center, VU University, 1081 HV Amsterdam, The Netherlands; d.meijer2@amsterdamumc.nl (D.M.); a.vis@amsterdamumc.nl (A.N.V.); 2 Department of Radiology and Nuclear Medicine, Cancer Center Amsterdam, Amsterdam University Medical Center, Location VUmc, 1081 HV Amsterdam, The Netherlands; m.cysouw@amsterdamumc.nl (M.C.F.C.); nh.hendrikse@amsterdamumc.nl (N.H.H.); r.boellaard@amsterdamumc.nl (R.B.); d.oprea-lager@amsterdamumc.nl (D.E.O.-L.)

**Keywords:** prostate cancer, prostate-specific membrane antigen, PET/CT, Theranostics

## Abstract

**Simple Summary:**

Imaging plays a crucial role in the accurate staging of prostate cancer. Prostate-specific membrane antigen (PSMA) is overexpressed in prostate cancer cells, and targeting the PSMA protein for diagnostic purposes has become of great clinical value. Another valuable feature of PSMA is its opportunity to serve as a target for delivering radionuclide therapy to cancer cells. PSMA-ligands can be labeled with various radionuclides, such as alpha and beta-emitters. This review offers an overview of the literature on recent developments in nuclear medicine regarding PSMA in prostate cancer diagnostics and targeted radionuclide therapy.

**Abstract:**

Targeting the prostate-specific membrane antigen (PSMA) protein has become of great clinical value in prostate cancer (PCa) care. PSMA positron emission tomography/computed tomography (PET/CT) is increasingly used in initial staging and restaging at biochemical recurrence in patients with PCa, where it has shown superior detection rates compared to previous imaging modalities. Apart from targeting PSMA for diagnostic purposes, there is a growing interest in developing ligands to target the PSMA-protein for radioligand therapy (RLT). PSMA-based RLT is a novel treatment that couples a PSMA-antibody to (alpha or beta-emitting) radionuclide, such as Lutetium-177 (^177^Lu), to deliver high radiation doses to tumor cells locally. Treatment with ^177^Lu-PSMA RLT has demonstrated a superior overall survival rate within randomized clinical trials as compared to routine clinical care in patients with metastatic castration-resistant prostate cancer (mCRPC). The current review provides an overview of the literature regarding recent developments in nuclear medicine related to PSMA-targeted PET imaging and Theranostics.

## 1. Introduction

Prostate cancer (PCa) is the second-most common malignancy worldwide, and it is the fifth leading cause of cancer-related mortality among men [1]. When detected at an early stage, patients tend to have an excellent prognosis. However, the course of PCa is heterogeneous and varies from indolent to highly aggressive disease [2,3]. Therefore, accurate staging and risk stratification are essential in the management of patients with PCa, given the wide variety of therapeutic options that may differ per disease stage. 

Currently, imaging plays a pivotal role in assessing the disease extent, particularly through targeting the prostate-specific membrane antigen (PSMA) [4]. PSMA is a transmembrane glycoprotein substantially overexpressed in malignant prostate cells [5]. As a result, PSMA is an attractive target for molecular imaging with positron emission tomography (PET) using one of several available radiolabeled PSMA-ligands. However, the expression of PSMA is not restricted to prostate (cancer) cells only and may be found in several non-prostatic diseases [5,6]. In clinical practice, the main indications to perform PSMA PET/computed tomography (CT) are initial staging and restaging at the biochemical recurrence of disease after treatment with curative intent [3,7,8]. Recently, the E-PSMA reporting guidelines have been proposed in order to harmonize protocols and to standardize PSMA PET/CT imaging reporting in PCa [9].

Aside from targeting the PSMA protein for diagnostic purposes, there is an increasing interest in using PSMA-radioligands for therapeutic purposes. This approach is called radioligand therapy (RLT). PSMA-RLT combines PSMA-ligands and therapeutic radionuclides to deliver targeted high radiation doses to cancer cells, leading to cellular death. PSMA-ligands can be labeled with either alpha (e.g., Actinium-225 (^225^Ac), Lead-212) or beta-emitting radionuclides (e.g., Lutetium-177 (^177^Lu)), with both having different characteristics in terms of physics and radiobiology [10]. Most experience has been gained with ^177^Lu-PSMA-617 in patients with metastatic castration-resistant prostate cancer (mCRPC) [11,12]. In the VISION trial, treatment with ^177^Lu-PSMA-617 resulted in an overall survival (OS) benefit of 4 months compared to routine clinical care [12]. 

This review summarizes the current literature on the recent developments in nuclear medicine regarding PSMA in PCa diagnostics and targeted radionuclide therapy. 

## 2. Prostate Cancer

### 2.1. Prostate Cancer Diagnosis

PCa suspicion rises with an abnormal digital rectal examination (DRE), an elevated serum prostate-specific antigen (PSA)-value, or both. However, PSA is organ-specific, not PCa specific, and might be increased in patients with benign diseases (i.e., prostatitis or benign prostate hyperplasia). Consequently, histopathological tissue assessment of prostate biopsies is required to confirm the diagnosis and estimate its aggressiveness, classified using the Gleason score (GS) [13]. Nevertheless, prostate biopsies are vulnerable to sampling errors, leading to false-negative outcomes and potentially inaccurate tumor evaluation [14]. Therefore, current international guidelines recommend multiparametric magnetic resonance imaging (mpMRI) in patients with an elevated PSA before prostate biopsy, allowing the targeted biopsy of suspicious radiological lesions [3]. Additionally, MRI provides essential information for local staging and planning of curative treatment, such as radical prostatectomy or radiation therapy [3]. Recently, the use of PSMA PET/CT for the initial staging of patients with high-risk PCa has also been recognized based on the results of several prospective studies [15,16].

### 2.2. Risk-Stratification and Local Tumor Staging

According to the International Society of Urological Pathology (ISUP) 2014, grading systems based on the GS, prostate biopsies are classified into five different grades groups of malignancy, ranging from 1 to 5 [13]. Alongside, the Tumor-Node-Metastasis (TNM) classification system is utilized for the uniform staging of PCa [17]. PCa is classified as an organ-confined (T1 and T2) or locally advanced disease (T3 and T4), the latter indicating that the tumor extends beyond the prostate and may invade adjacent structures. These clinical parameters (i.e., TNM stage, PSA, and ISUP grade) are implemented in the European Association of Urology (EAU) PCa risk categories, dividing patients into low, intermediate, or high-risk disease groups [3]. Higher risk groups are associated with an increased risk of having or developing metastatic disease. This underlines the essence of correct and complete staging in these patients, including assessment of metastatic dissemination. 

### 2.3. Staging of Metastases in Prostate Cancer

The assessment of regional lymph node metastases (N-status) and distant metastases (M-status) is crucial for the accurate staging of patients with PCa since it affects therapy planning and prognosis. Unfortunately, the median survival of men with newly diagnosed metastatic (M1) PCa is approximately 42 months [18]. Common metastatic sites are local and/or distal lymph nodes and bone, while visceral metastases occur less frequently. According to the EAU guidelines, metastasis screening at initial diagnosis is recommended in intermediate and high-risk disease by at least abdominopelvic imaging and bone scintigraphy (BS) [3]. However, the diagnostic accuracy of these conventional imaging modalities is limited for detecting PCa lesions [19,20]. For example, the sensitivity of CT and MRI for pelvic lymph node detection is only 42% and 39%, respectively [19]. A potential explanation may be that these imaging modalities primarily rely upon lesion morphology (i.e., the shape and size of a lesion) for detection, which might be inaccurate in (early) metastatic PCa with small metastases being missed.

## 3. PSMA PET Diagnostics

Radiolabeled PSMA-ligands have recently been introduced to the rapidly evolving nuclear imaging field. While most studies have investigated its performance in either primary staging or restaging at biochemical recurrence (e.g., rising PSA after local therapy), there is increasing data regarding its use in the follow-up of patients with mCRPC. PSMA-ligands can be labeled with ^68^Gallium (^68^Ga) or ^18^Fluoride (^18^F). ^18^F-labeled tracers have increased positron yield and shorter positron range compared with ^68^Ga-labeled tracers, resulting in a higher resolution of the images, with potentially enhanced detection of (small) metastases. Additionally, ^18^F has the advantages of a longer half-life (110 versus 68 min for ^68^Ga), enabling centralized production on a larger scale [21]. ^68^Ga-PSMA-11 and ^18^F-DCFPyL are the most commonly used radioligands and are primarily excreted by the urinary tract, often making the interpretation of the prostate bed and/or metastases adjacent to the ureters challenging [4,22]. A relatively novel introduced ^18^F-labeled tracer is ^18^F-PSMA-1007, with a comparable diagnostic accuracy as ^68^Ga-PSMA-11 and ^18^F-DCFPyL for detecting the local recurrence of PCa in the prostatic fossa [23,24,25,26]. The ^18^F-PSMA-1007 excretion pathway is mainly by the hepatobiliary tract and marginally by urinary excretion, yielding the potential benefit to differentiate nodal metastases or local recurrence from physiological urinary activity [23,24,27]. A disadvantage of ^18^F-PSMA-1007 is its high unspecific bone uptake, leading to a greater prevalence of positive PSMA findings attributed to a benign origin. Therefore, extensive reader training is necessary to become familiar with the interpretation and reporting [25,26]. Implementing the recently developed E-PSMA criteria might mitigate these clinically relevant interpretation differences among readers in routine daily practice [9].

### 3.1. Initial Staging

Recent studies have demonstrated the advantages of PSMA PET/CT in the primary staging of men with PCa compared to conventional imaging modalities [8,16,19,20,28]. For example, Pienta et al. evaluated the performance of ^18^F-DCFPyL, a second-generation PSMA-ligand PET/CT, in detecting metastatic disease at initial staging in high-risk PCa compared with histopathology in the OSPREY trial. In this prospective multicenter phase II/III trial, a total of 252 patients with high-risk PCa planned for radical prostatectomy with lymph node dissection were included. ^18^F-DCFPyL PET/CT compared to CT or MRI alone showed higher specificity (97.9% versus 65.1%, respectively), positive predictive value (PPV) (86.7% versus 28.3%, respectively), and negative predictive value (NPV) (83.2% versus 77.8%, respectively), with similar sensitivity (40.3% versus 42.6%, respectively) for the detection of pelvic lymph node involvement (LNI) [8]. Similar results were found when investigating the diagnostic accuracy of ^68^Ga-PSMA and ^18^F-DCFPyL PET/CT for lymph-node staging in primary PCa [29,30]. The prospective cohort study by van Kalmthout et al. reported a limited sensitivity (41.5%) and high specificity (90.9%) for detecting pelvic lymph node metastases with ^68^Ga-PSMA PET/CT in patients with newly diagnosed PCa [30]. A similar study from Jansen et al. reported a sensitivity and specificity of 41.2% and 94.0%, respectively, for detecting lymph node metastases with ^18^F-DCFPyL PET/CT [29]. Nevertheless, mainly based on the encouraging results from the ‘proPSMA’ trial, the European Association of Urology (EAU) guidelines have recently incorporated PSMA PET/CT for initial staging purposes [3]. In this prospective multi-center study, 302 patients with high-risk PCa, prior to curative-intent surgery or radiotherapy, were randomly assigned to conventional imaging with CT and bone scintigraphy or ^68^Ga-PSMA-11 PET/CT. The accuracy of ^68^Ga-PSMA PET/CT was 27% higher than that of CT and bone scintigraphy (92% versus 65%; *p* < 0.0001). Conventional imaging had a lower sensitivity (38% versus 85%) and specificity (91% versus 98%) than PSMA PET/CT. Moreover, the ^68^Ga-PSMA PET/CT scan induced management change more frequently than conventional imaging, with less equivocal findings and lower radiation exposure [16].

A PSMA PET/CT limitation is that a negative PSMA PET/CT cannot rule out lymph node metastases [8,29,30,31]. Consequently, the ePLND remains the gold standard for primary nodal staging, despite known potential complications, such as lymphocele, lymphedema, and deep venous thrombosis [3].

### 3.2. Biochemical Persistence

In 5–20% of the patients treated with radical prostatectomy (RP), the PSA level remains measurable after treatment [32,33]. Biochemical persistence (BCP) is defined as a detectable PSA level of ≥0.1 ng/mL within 4–6 after RP [34]. Causes of BCP are the presence of (micro)metastases or residual disease in the prostatic tissue. Unfortunately, BCP is associated with more advanced PCa, such as higher pathological tumor stages, higher ISUP grade, positive surgical margins, and an impaired prognosis [33,35,36]. Schmidt-Hegemann et al. more frequently observed pelvic LNI on ^68^Ga-PSMA PET/CT in patients with BCP than patients who develop biochemical recurrence [37]. The multicenter retrospective study by Farolfi et al. reported that ^68^Ga-PSMA PET/CT localized PCa in two-thirds of the patients with BCP [38]. Additionally, Meijer et al. analyzed the findings of ^68^Ga-PSMA PET/CT and ^18^F-DCFPyL PET in 150 patients with BCP after surgical treatment. They found PSMA positive lesions outside the prostatic fossa in 67% of the patients and in 26% of patients outside the pelvis [39]. Therefore, accurate localization of residual disease with PSMA PET/CT is critical to determine and guide the most effective treatment. 

### 3.3. Restaging at Biochemical Recurrence

PSMA PET/CT has been extensively evaluated in patients with biochemically recurrent disease (BCR) after definite treatment. BCR is defined as a serum PSA of ≥0.2 ng/mL after radical prostatectomy or a serum PSA ≥ 2.0 ng/mL above the nadir after radiation therapy [40,41]. In patients with BCR, identifying the recurrence site is crucial as it directly influences therapeutic decision-making. The detection of metastatic disease is strongly associated with the level of PSA-values when performing the PSMA PET/CT [7,28,42]. Interestingly, Jansen et al. analyzed PSMA PET/CT performed in 63 patients with low PSA levels (<2.0 ng/mL, not meeting BCR criteria) after curative radiotherapy and found PSMA positive lesions in 53/63 patients (84.1%) defined as local recurrence (21 patients) or metastatic disease (32 patients) [43]. Perera et al. reported sensitivities for ^68^Ga-PSMA PET/CT in detecting BCR of 33%, 45%, 59%, 75%, and 95% for PSA ranges of <0.2, 0.2–0.49, 0.5–0.99, 1.0–1.99, and ≥2.0 ng/mL, respectively [28]. 

Before the introduction of PSMA PET, prostate cancer molecular imaging was commonly performed using radiolabeled choline-ligands (e.g., ^11^C-choline and ^18^F-choline) and more recently ^18^F-Fluciclovine [15,44,45,46,47]. In the literature, ^68^Ga-PSMA PET/CT has demonstrated higher detection rates than ^11^C-Choline PET/CT in BCR, especially in patients with low PSA levels [44,45,46,47,48]. A recent prospective trial by Calais et al. enrolled 50 patients with BCR after RP with low a PSA level (<2.0 ng/mL) to compare the detection rate and reproducibility of ^68^Ga-PSMA PET/CT versus ^18^F-Fluciclovine. They found significantly higher detection rates with ^68^Ga-PSMA PET/CT compared to ^18^F-Fluciclovine (56% versus 26%; OR 4.8 95%CI: 1.6–19.2, *p* = 0.0026), also when stratified by PSA level (PSA < 0.5 ng/mL: 46% versus 27%; PSA 0.5–1.00 ng/mL: 67% versus 28%; PSA 1.01–2.00: 67% versus 17%, respectively) [15]. Furthermore, the recent prospective, phase III CONDOR trial by Morris et al. assessed the diagnostic performance of ^18^F-DCFPyL in patients with BCR with negative or equivocal findings on PET/CT (^18^F-Fluciclovine or ^11^C-Choline) or conventional imaging (CT, MRI, or BS). Improved detection rates were found when PSA levels were higher (PSA < 0.5 ng/mL: 36.2%; PSA 0.5–0.99 ng/mL: 51.4%; PSA 1.0–1.99: 66.7%). A high correct localization rate (84.8–87.0% lower bound of 95%CI: 77.8–80.4) was found. Furthermore, disease management was changed in nearly two-thirds of the analyzed patients (63.9%, *n* = 131) [7]. 

**Figure 1:**
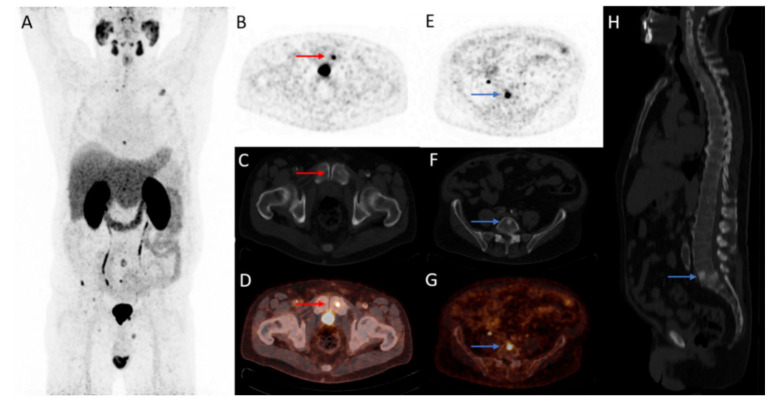
A 70-year-old patient with biochemical recurrence after radical prostatectomy (Gleason 3 + 4 = 7, PSA-nadir < 0.1 ng/mL) with a PSA of 0.7 ng/mL at the PET/CT scan time. Restaging ^18^F-DCFPyL PET/CT detected multiple bone metastases (>10) at low serum PSA value ((**A**); maximum intensity projection). Transversal ^18^F-DCFPyL PET (**B**,**E**) and fused PET/CT (**D**,**G**) images illustrate two bone metastases (os pubis left, red arrow: SUV_max_: 9.76; L5 vertebra, blue arrow SUV_max_: 8.02) with sclerotic substrate on CT (**C**,**F**,**H**).

Apart for cohort A of the phase 2/3 OSPREY trial, cohort B included patients with suspected locoregional recurrence and/or distant metastatic disease on conventional imaging (CT, MRI, or BS). Among all patients, high median sensitivity (95.8%) and PPV (81.9%) of ^18^F-DCFPyL PET/CT were found for detecting recurrence or metastatic disease, respectively. Moreover, metastatic disease was described in 57.6% of the patients previously staged with locoregional disease on conventional imaging. The sensitivity ranged from 88.9% to 100% and the PPV from 61.5% to 88.9% in patients with low PSA levels (<2.0 ng/mL) [8]. Considering these superior detection rates of PSMA PET/CT on biochemical recurrence of disease, PSMA PET/CT has become the recommended imaging modality for BCR following previous curative-intent therapy (Figure 1) [3]. 

PSMA PET/CT is increasingly used to select the optimal treatment strategy in patients with BCR, and PSMA PET findings frequently result in management changes [49,50,51]. For example, Meijer et al. found a change of preferred management in 40.7% of the patients with BCR who underwent ^18^F-DCFPyL PET/CT for restaging after curative-intent treatment [50]. Likewise, Calais et al. assessed the impact of ^68^Ga-PSMA PET/CT on the treatment plan of BCR and showed a change of management in 53% of the patients [49]. 

When PCa recurrence is restricted to the prostatic fossa, salvage radiation therapy (SRT) may be considered as a potentially curative treatment option and proves to be the most effective at a PSA value of ≤0.5 ng/mL [34]. However, the findings on PSMA PET/CT before SRT impact the planned treatment by extending the target volume, implying dose escalations, or refraining from radiotherapy [52,53,54]. Since the introduction of PSMA PET/CT, patients with BCR may be diagnosed as having metastatic disease at an earlier stage, also known as ‘stage migration’. Patients with the oligometastatic disease have a limited number of metastases (usually defined as 1–5 metastatic lesions). Metastasis-directed radiotherapy (MDT) on these lesions may postpone the initiation of systemic treatment [55,56,57]. A phase II randomized clinical trial by Philips et al. compared stereotactic body radiation therapy (SBRT) observation in patients with oligometastatic recurrent PCa (up to three metastases) on conventional imaging. ^18^F-DCFPyL PET/CT was performed at baseline in the patients receiving SBRT, and these results were blinded to the investigative team during therapy planning. A higher number of patients progressed at six months in the observational cohort than into the group allocated to SBRT (61% versus 19%). The SBRT treatment plan was compared to the results of the PSMA PET/CT, and patients were divided into a total and subtotal consolidation of PSMA avid lesions. Total consolidation of PSMA lesions decreased the risk of new lesions at six months (16% versus 63%) [57]. This study highlights the impact of PSMA PET/CT in planning MDT in patients with oligometastatic disease. However, the long-term effect on overall survival and quality of life are still to be demonstrated. 

### 3.4. Castration-Resistant Prostate Cancer

**Figure 2:**
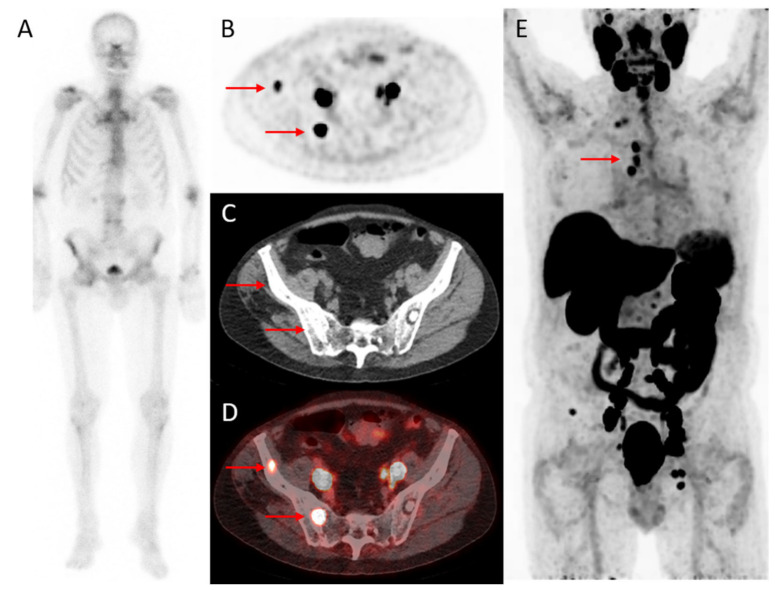
A 79-year-old patient with CRPC after initial treatment with radiotherapy followed by hormonal therapy. Images illustrate improved detection of bone metastases using ^18^F-DCFPyL PET/CT compared to bone scintigraphy (4 weeks interval). The PSA level at PET was 23 ng/mL. On bone scintigraphy, faint uptake in the lumbar spine, the right acromioclavicular joint, the sternoclavicular, and hip joints were attributed to degenerative changes (**A**). Transversal ^18^F-DCFPyL PET (**B**) and fused PET/CT (**D**) revealed two foci (red arrows) with intense PSMA-expression in the right iliac bone (SUV_max_: cranial lesion 6.2 and caudal lesion 17) and a sclerotic substrate on CT (**C**) and were classified as highly suspicious for bone metastases. Maximum intensity projection (**E**) demonstrated additional lymph node metastases above the diaphragm.

Castration-resistant prostate cancer (CRPC) is defined as biochemical or radiological progression of disease on conventional imaging in the presence of castration levels of serum testosterone (i.e., <50 ng/dL) [34,58]. In CRPC, the number of available therapeutic choices has increased, while the optimal treatment strategy is not fully established [34,59,60,61,62,63]. Current guidelines (PCWG3 and EAU) recommend conventional imaging in combination with regular blood tests for staging and evaluating disease progression in mCRPC patients, but their sensitivity is known to be limited (Figure 2) [34,58]. 

For example, the multicenter retrospective study of Fendler et al. was designed to assess ^68^Ga-PSMA PET performance in CRPC patients without metastases on conventional imaging. Distant metastatic disease was found in 55% of the included patients [64]. More sensitive detection with PSMA PET, and potentially earlier detection of metastatic disease, could impact the course of the disease and may facilitate the initiation of early treatment or timely therapy switch to another therapy [65]. However, the resulting improvement in oncological outcomes has not yet been demonstrated.

PSMA PET/CT could be performed for selecting patients for PSMA-directed RLT and (re)staging during or after treatment. It is essential to assess the level of PSMA expression before initiating RLT, as PSMA expression in mCRPC disease is known to be highly variable both within and between patients [66]. As a consequence, approximately one-third of the patients will not respond to PSMA-RLT. Hence, identifying predictors of treatment response could be of great value [67]. Ferdinandus et al. described that a higher platelet level and need for pain medication were significant predictors of a poor treatment response to ^177^Lu-PSMA-617, and PSMA expression on ^68^Ga-PSMA PET/CT did not predict PSA response [68]. In a similar cohort, Emmett et al. aimed to identify predictors of treatment response in mCRPC patients treated with ^177^Lu-PSMA-617. They found a strong correlation of PSMA expression (standardized uptake value (SUV): SUV_max_ and SUV_mean_) on ^68^Ga-PSMA PET/CT at baseline imaging with a treatment response of more than 30%. The location or volume of metastases were no predictors of treatment response [69]. 

### 3.5. Reporting PSMA PET/CT

In recent years, a variety of reporting systems have been provided, including staging and lesion characterization, to improve consistent PSMA PET/CT describing [70,71]. Furthermore, the newly proposed E-PSMA consensus guidelines, endorsed by the European Association of Nuclear medicine, offers PSMA PET/CT interpretations and reporting statements to create more uniform and standardized reports for clinical use [72]. These guidelines incorporate earlier proposed PSMA-RADS (PSMA-reporting and Data system) and PROMISE (Prostate Cancer Molecular Imaging Standardized Evaluation) criteria. The PSMA-RADS categorizes PSMA PET/CT findings into five categories based on the probability of malignancy [71]. Furthermore, the PROMISE criteria include the intensity of PSMA expression (ranging from 0–3) and the molecular imaging TNM scores (miTNM score) [70]. Recently, a deep learning algorithm (aPROMISE) has been developed for the automated analysis of PSMA PET images to provide a consistent and standardized evaluation. However, the results of the aPROMISE technology require further validation before it can be translated into clinical practice [73].

## 4. Theranostics

### 4.1. PSMA-Radioligand Therapy

Aside from targeting PSMA for diagnostic purposes, another valuable feature of PSMA is its opportunity to serve as a target for delivering radionuclides (therapeutic agents) to cancer cells. Using the same target for diagnosis and therapeutics is referred to as Theranostics. Recently, novel radionuclides have been developed and proposed to be used as RLT in clinical practice for PCa management. For example, PSMA-ligands can be labeled with varying radionuclides, such as alpha and beta-emitters [10]. The most frequently used radionuclides for PSMA-RLT are Lutetium-177 (^177^Lu), which decays by beta-emission, and Actinium-225 (^225^Ac), alpha-emission. 

There are several clinically relevant differences between alpha and beta-particles (Table 1) [10]. Alpha-particles have a larger mass and carry higher energies. Alpha-particles have high linear-energy transfer (LET), defined as the amount of energy a particle can transmit along its track. This leads to more damage down their track and causes irreparable double-strand DNA breaks in tumor cells. Alpha-particles have a limited range in tissue (0.05–0.08 mm), providing more controlled and selective irradiation of cancer cells with minimal impact on neighboring tissue [10,74,75]. In contrast, beta-particles have a small mass and a more extended range in tissue (0.62 mm). However, they have less energy in comparison with alpha particles. The LET produced by beta-particles is relatively low, resulting in single-strand DNA breaks, which are repairable and thus may be less effective in damaging PCa cells [10,75]. However, the advantage of the beta-emitter, ^177^Lu-PSMA is its favorable toxicity profile with less severe side-effects. 

### 4.2. Beta-Emitter Radio-Ligand Therapy: Lutetium-PSMA

PSMA-617 is the most commonly used ligand in RLT, which can be coupled to Lutetium-177, resulting in ^177^Lu-PSMA-617 [76]. In addition, ^177^Lu can also be attached to the PSMA Imaging and Therapy ligand (^177^Lu-PSMA I&T) [77]. However, the use of ^177^Lu-PSMA-617 might be preferred in clinical practice compared to ^177^Lu-PSMA I&T, possibly due to reduced uptake in the kidney [78]. RLT with ^177^Lu-PSMA has mainly been studied in mCRPC, showing promising results as a potential treatment approach with a low toxicity profile [11,12,67,79,80,81,82,83]. 

Several retrospective studies have outlined the biochemical (PSA) response of ^177^Lu-PSMA-617 in mCRPC (see also Table S1) [84,85,86,87,88,89,90]. Kratochwil et al. reported any PSA response from baseline in 21 (70%) of 30 patients, and a PSA decline of more than 50% was found in 43% (13/30) after ^177^Lu-PSMA-617 treatment [88]. Similarly, in a study including 100 mCRPC patients with a history of treatment with enzalutamide or abiraterone, Ahmadzadehfar et al. reported any PSA decline and a PSA decline of >50% in 69% and 38% after ^177^Lu-PSMA-617 therapy [84]. In another study, Ahmadzadehfar et al. evaluated the patient response to the second and third cycle of ^177^Lu-PSMA-617 in 52 patients and found PSA decline > 50% in 60% of the patients [85]. In a retrospective study of Brauer et al., any PSA decline was found in 91% of the patients (*n* = 45), and a PSA reduction of greater than 50% occurred in 53%. Any PSA decline after the first treatment cycle was significantly associated with a longer OS [86]. Rahbar et al. included patients with mCRPC treated with ^177^Lu-PSMA-617 to assess the efficacy and safety of ^177^Lu-PSMA-617. A PSA decline of 50% or more was found in 45% of the patients. Grades 3 and 4 hematotoxicity occurred in 12% of the patients, and xerostomia was reported in 8% [89]. Another recent publication on ^177^Lu-PSMA-617 conducted by Rahbar et al. recorded any PSA response in 67% of the 104 included men and a PSA decline of >50% in 33%. Any PSA decline after the first cycle was associated with a longer OS than PSA progression (62.9 versus 47.0 weeks). A PSA decline greater than 50% was not prognostic for overall survival [90].

Hofman et al. conducted a single-center, phase II trial including mCRPC patients with progressive disease after conventional treatment. Treatment with ^177^Lu-PSMA-617 treatment resulted in any PSA level decline in 97% of the patients and a PSA decline of ≥50% in 57%. Most registered adverse events (AE) were xerostomia grade I (87%), transient nausea (50%), and fatigue grade I–II (50%). Grade 3–4 thrombocytopenia due to ^177^Lu-PSMA-617 occurred in 13% of the patients [80].

The randomized, multicenter, phase II TheraP trial compared ^177^Lu-PSMA-617 (up to six cycles every six weeks) to cabazitaxel (up to 10 cycles every three weeks) in 200 patients with progressive post-docetaxel mCRPC. Patients treated with ^177^Lu-PSMA-617 showed a ≥50% PSA response more frequently than patients treated with cabazitaxel (66% versus 37%, *p* < 0.0001). In addition, fewer grade III and IV AE were observed in patients who underwent ^177^Lu-PSMA-617 treatment (33% versus 53%) [11]. 

Furthermore, the randomized, phase III VISION trial by Sartor et al. assessed 831 patients with mCRPC diagnosed with at least one positive lesion on ^68^Ga-PSMA-11 PET/CT. The patients previously underwent treatment with minimal one androgen receptor signaling pathway inhibitor and taxane chemotherapy. The patients were randomized 2:1 to receive ^177^Lu-PSMA-617 (every six weeks up to four–six cycles) plus standard of care (SOC; (*n* = 551) or SOC alone (*n* = 280). The median imaging-based progression-free survival was improved by 5.3 months in the ^177^Lu-PSMA-617 group compared to the control group (8.7 versus 3.4 months, respectively; *p* < 0.001). In addition, there was a significant median OS benefit in favor of ^177^Lu-PSMA-617 (15.3 versus 11.3 months, respectively; *p* < 0.001). As expected, treatment with ^177^Lu-PSMA-617 led to a higher incidence of grade 3 AE, or higher, than the control group (52% versus 38%). The most-reported AE were fatigue, dry mouth, and nausea grade I or II. Nevertheless, a low incidence of AE led to alternation of the doses or discontinuation of the study, and treatment with ^177^Lu-PSMA-617 was considered safe [12]. Challenges remain in the prediction of treatment response and survival in ^177^Lu-PSMA therapy. In several studies, (changes in) metrics quantifying the burden of PSMA-positive disease on PET were associated with treatment response and survival to ^177^Lu-PSMA radioligand therapy in patients with mCRPC [91,92,93] 

There is increasing interest in positioning PSMA-radioligand therapy in the (earlier) hormone-sensitive stage. It is hypothesized that in metastatic hormone-sensitive prostate cancer (mHSPCa), the initiation of androgen deprivation therapy (ADT) can be deferred, and, ultimately, the OS could be improved. Several studies are ongoing in patients with mHSPCa, and results are eagerly awaited [NCT04443062; NCT04343885; NCT04720157]. 

### 4.3. Alpha-Emitter Radioligand Therapy: Actinium-PSMA

The most commonly used alpha-emitter for PSMA-ligand treatment is ^225^Ac-PSMA-617 (see also Table S2). A retrospective study by Kratochwil et al. included 40 patients with mCRPC who underwent treatment with ^225^Ac-PSMA-617 (every two months up to three cycles). In total, 63% of patients had a PSA decline of more than 50%, and 87% had any PSA response. Remarkably, five patients (13%) showed a response for over two years. Unfortunately, four patients (10%) dropped out of this study because of (severe) side effects (xerostomia), and five patients (13%) terminated treatment due to lack of response following the first cycle [94]. Sathekge et al. enrolled 73 patients with mCRPC for treatment with ^225^Ac-PSMA-617 (every eight weeks, most patients received up to two–five cycles). A total of 82% of patients had any PSA response in this cohort, and 70% had a PSA decline of >50%. Grades I and II xerostomia were reported in 85% of the patients, not leading to treatment discontinuation [95]. 

^225^Ac-PSMA-617 could benefit patients who did not respond to prior ^177^Lu-PSMA-RLT. Several studies included patients previously treated with ^177^Lu-PSMA-RLT. Yadav et al. prospectively enrolled 28 men with mCRPC to receive ^225^Ac-PSMA-617 treatment (median of three cycles). A total of 54% of these had prior exposure to ^177^Lu-PSMA therapy. After the first treatment cycle, 25% of the patients had a PSA decline of ≥50%, which increased to 39% at the end of follow-up. Any PSA decline was found in 78.6%. Patients’ refractory to ^177^Lu-PSMA less frequently showed a PSA decline of ≥50% than patients with no history of ^177^Lu-PSMA therapy (26.6% versus 53.8%). Half of the patients reported fatigue and 29% xerostomia (grade I/II) as AE [96]. In the study by Fuerecker et al., ^225^Ac-PSMA-617 was offered every eight weeks (median of two cycles) to 26 patients with mCRPC who progressed after a median of four cycles of ^177^Lu-PSMA treatment. In 88% of the patients, any PSA decline was described, and 65% had a PSA decline of ≥50%. Grade I/II xerostomia was observed in all patients, leading to study discontinuation in six patients (23%). The reported hematological AE (grade III/IV) were thrombocytopenia (19%), leucopenia (27%), and anemia (35%) [97]. Although these retrospective studies seem promising, further prospective data is warranted. Unfortunately, the clinical application of ^225^Ac-PSMA RLT is sparse due to the limited availability of ^225^Ac [98].

## 5. Conclusions

In recent years, PSMA PET has gained an increasingly important role in both initial diagnosis and at the biochemical recurrence of disease in patients with prostate cancer. In addition, PSMA PET/CT is being used more frequently during follow-up of the disease to assess treatment response. Aside from targeting the PSMA protein for diagnostic purposes, PSMA may also be a target for combined diagnostics and therapeutic purposes, the Theranostics approach. PSMA radioligand therapy has shown to be an effective and safe therapeutic option for patients with metastatic castration-resistant prostate cancer. Its oncological effect is currently being investigated in patients presenting with metastatic hormone-sensitive prostate cancer.

## Figures and Tables

**Table 1:** Radionuclide properties of Actinium-225 and Lutetium-177. Reference: Sgouros G, Nature reviews (2020); 589–608 [10].

Radionuclide Property	Actinium-225	Lutetium-117
Therapeutic emission	α	β−
Emission in range in tissue (mm)	0.05–0.08	0.62
Radionuclide half-life (days)	10.0	6.6

## Data Availability

No new data were created or analyzed in this study. Data sharing is not applicable to this article.

## Supplementary Materials

The following supporting information can be downloaded at: https://www.mdpi.com/article/10.3390/cancers14051169/s1, Table S1: Summary of 177Lu-PSMA-617 studies; Table S2. Summary of ^225^Ac-PSMA-617 studies.
