# Supplementary material for: Targeting PSMA Revolutionizes the Role of Nuclear Medicine in Diagnosis and Treatment of Prostate Cancer
Source: Cancers (Basel). 2022 Feb 24;14(5):1169. doi: 10.3390/cancers14051169 (PMC8909566; doi:10.3390/cancers14051169)
Supplement: Supplementary file 1 [file cancers-14-01169-s001.zip › cancers-1613865-supplementary.pdf]

**Supplementary material**

**Table S1**. Summary of $^{177}$Lu-PSMA-617 studies

| **First author** | **Year** | **RLT Agent** | **Study type** | **Number of patients treated** | **Frequency of PSA decline of ≥50%** |
|---|---|---|---|---|---|
| Fendler | 2016 | $^{177}$Lu-PSMA-617 | Retrospective | 15 | 60 (9/15) |
| Kratochwil | 2016 | $^{177}$Lu-PSMA-617 | Retrospective | 30 | 43 (13/30) |
| Ahmadzadehfar PO | 2017 | $^{177}$Lu-PSMA-617 | Retrospective | 100 | 38 (38/100) |
| Ahmadzadehfar overall l | 2017 | $^{177}$Lu-PSMA-617 | Retrospective | 52 | 60 (31/52) |
| Brauer | 2017 | $^{177}$Lu-PSMA-617 | Retrospective | 45 | 53 (24/45) |
| Rahbar | 2017 | $^{177}$Lu-PSMA-617 | Retrospective | 145 | 45 (45/99) |
| Rahbar | 2018 | $^{177}$Lu-PSMA-617 | Retrospective | 104 | 33 (34/104) |
| Hofman *LuPSMA* | 2018 | $^{177}$Lu-PSMA-617 | Phase II clinical trial | 30 | 57 (17/30) |
| Hofman *TheraP trial* | 2021 | $^{177}$Lu-PSMA-617 | Phase II clinical trial | 98 | 66 (65/99) |
| Sartor *Vision trial* | 2021 | $^{177}$Lu-PSMA-617 | Phase III clinical trial | 385 | 46 (177/385) |

**Table S2.** Summary of $^{225}$Ac-PSMA-617 studies

| First author | Year | RLT Agent | Study type | Number of patients treated | Frequency of PSA decline of ≥50% |
|---|---|---|---|---|---|
| Kratochwil | 2018 | **$^{225}$Ac-PSMA-617** | Retrospective | 40 | 63 (24/38) |
| Sathekge | 2020 | **$^{225}$Ac-PSMA-617** | Retrospective | 73 | 70 (51/73) |
| Yadav | 2020 | **$^{225}$Ac-PSMA-617** | Prospective | 28 | 39 (11/28) |
| Fuerecker | 2021 | **$^{225}$Ac-PSMA-617** | Retrospective | 26 | 65 (17/26) |
